# Modulation of oxidative stress and plant responses to salinity by nanosilicon: current insights and future perspectives

**DOI:** 10.3389/fpls.2026.1857009

**Published:** 2026-07-03

**Authors:** Neda Nikpour Rashidabad, Salar Farhangi-Abriz, Masoud Hashemi

**Affiliations:** 1 Stockbridge School of Agriculture, University of Massachusetts Amherst, Amherst, MA, United States; 2 Cotton Research Institute of Iran, Agricultural Research, Education and Extension Organization (AREEO), Gorgan, Iran

**Keywords:** antioxidant defense, nanosilicon, oxidative stress, ROS, salt stress

## Abstract

Soil salinity is a formidable challenge to global food security, triggering severe oxidative stress and reactive oxygen species (ROS) overproduction that devastate crop productivity. Nanosilicon (1–100 nm) has recently emerged as a transformative, highly reactive elicitor capable of counteracting these detrimental effects more efficiently than conventional bulk silicon. This comprehensive review critically evaluates the underlying mechanisms of nanosilicon-mediated salt tolerance and its practical implications for sustainable agriculture. By offering superior cellular penetration and bioavailability, nanosilicon mitigates ROS, such as superoxide radicals (O_2_^−^) and hydrogen peroxide (H_2_O_2_), subsequently reducing lipid peroxidation by up to 50% across various crops. Beyond direct scavenging, it fortifies both enzymatic and non-enzymatic antioxidant defense systems and modulates stress-responsive gene networks via abscisic acid (ABA) and mitogen-activated protein kinase (MAPK) signaling cascades. By synergizing osmotic adjustment, ion homeostasis, and photosynthetic protection, these nanoscale interventions can drive yield improvements of up to 30% under saline conditions. Crucially, we address the current limitations, emphasizing that nanosilicon’s efficacy is highly dependent on plant species, particle size, and environmental variables. While challenges such as dose-dependent phytotoxicity, environmental risks, and production costs require further investigation, optimizing nanosilicon formulations holds profound potential for developing climate-resilient agriculture.

## 1 Introduction

Salinity is a major stressor that adversely impacts plant health, growth, and agricultural productivity, posing a major challenge to global food security. As soil salinity intensifies, affecting approximately 20% of cultivated land and 33% of irrigated agricultural land globally, due to factors such as climate change and poor agricultural practices, plants show a range of physiological and biochemical adaptations to cope with these stressful conditions (Hao et al., 2021; Ghassemi-Golezani and Farhangi-Abriz, 2021). A key challenge under salt stress is ionic toxicity, caused by the excessive accumulation of sodium (Na^+^) and chloride (Cl^−^) ions in plant tissues, disturbing vital cellular tasks (Ghassemi-Golezani and Nikpour-Rashidabad, 2017). The ion buildup disrupts essential cellular processes and interferes with nutrient uptake, ultimately harming plant growth and development (Farhangi-Abriz and Ghassemi-Golezani, 2021).

Salt-induced ionic and osmotic stress triggers the overproduction of reactive oxygen species (ROS), which can cause severe damage to cellular structures such as membranes, proteins, and DNA (Ondrasek et al., 2022). Consequently, this oxidative stress impairs key metabolic functions, notably photosynthesis and respiration, leading to chlorophyll degradation and reduced overall plant growth (Hasanuzzaman et al., 2021). To defend against such oxidative damage, plants rely on sophisticated endogenous antioxidant systems comprising enzymatic components, such as superoxide dismutase (SOD) and catalase (CAT), alongside non-enzymatic molecules like ascorbic acid and glutathione (Ghassemi-Golezani and Nikpour-Rashidabad, 2017; Fujita and Hasanuzzaman, 2022). However, under severe saline conditions, these natural defense mechanisms are often overwhelmed, creating a critical need for effective external interventions to bolster plant resilience. Although traditional bulk silicon has been widely applied to mitigate abiotic stresses, its low solubility and limited tissue uptake restrict its overall efficacy. This specific gap has driven recent attention toward nanoscale silicon, which, due to its unique physicochemical properties, higher surface-area-to-volume ratio, and enhanced cellular penetration, offers a far more efficient alternative for modulating stress signaling and reinforcing plant antioxidant defenses.

Nanosilicon (nSi) has emerged as a promising tool to enhance plant tolerance to salt stress through multiple physiological and biochemical mechanisms. One of the primary benefits of nSi is mitigating osmotic stress and maintaining ion homeostasis (Shoukat et al., 2024a; Ghassemi-Golezani et al., 2024). Under saline conditions, sodium ions compete with potassium ions for uptake, leading to nutrient imbalance and reduced nutrient acquisition. Nanosilicon facilitates the maintenance of a favorable K/Na ratio within plant tissues (Sabaa and Hashem, 2025) by improving potassium absorption and reducing sodium accumulation, thereby supporting vital physiological functions and improving plant growth in saline environments (Mahmoud et al., 2022).

Due to its small size and high surface area, nanosilicon penetrates plant tissues more efficiently than bulk silicon (Sarkar et al., 2022). It mitigates salt stress through several key mechanisms: restoring ionic balance by reinforcing cell walls and regulating ion transport (Dhiman et al., 2021); reducing ROS-induced oxidative damage via the upregulation of antioxidant enzymes like SOD, CAT, and APX (Farhangi-Abriz and Torabian, 2018); facilitating osmotic adjustment by accumulating compatible solutes such as proline and glycine betaine (Ismail et al., 2022); and modulating stress-responsive gene expression (Singh et al., 2025). Consequently, nanosilicon enhances growth, photosynthesis, and yield in crops such as wheat (Aouz et al., 2023), rice (Xiong et al., 2024), and tomato (Sayed et al., 2022). However, its overall efficacy depends on plant species, concentration, application methods, and environmental conditions, necessitating further research to optimize its use in sustainable agriculture (Dhiman et al., 2021).

This review aims to provide a comprehensive synthesis of current knowledge of nanosilicon in alleviating salt stress, with a particular emphasis on its influence on oxidative stress management. Unlike previous reviews that primarily focus on bulk silicon, this paper distinctly highlights the unique nanoscale properties of nanosilicon and its superior efficacy as both a physical barrier and a signaling modulator. The review will focus on how nanosilicon modulates plant responses to salinity, particularly examining its effects on ROS production, enhancing antioxidant defense systems, and influencing related biochemical and molecular responses (such as hormone and kinase signaling pathways). The review will also explore experimental findings across diverse plant species, highlighting both the potential and limitations of nanosilicon applications under saline conditions. In doing so, this review seeks to identify knowledge gaps, such as variability in nanosilicon efficacy across species and conditions, and propose directions for future studies to enhance its practical application in agriculture. By bridging scientific insights and agricultural needs, this review aims to underscore nanosilicon’s potential as an innovative approach to improving crop resilience in salt-affected regions, thereby contributing to global food security amid increasing soil salinization.

## 2 Oxidative stress under salt stress conditions

### 2.1 Generation of reactive oxygen species in plants

Under salt stress, the generation of ROS is markedly augmented due to disruptions in cellular redox homeostasis. To understand this phytotoxicity, it is crucial to separate primary ROS production sites from downstream amplification processes (Farhangi-Abriz and Ghassemi-Golezani, 2019; Sachdev and Ahmad, 2021). Primary ROS generation occurs predominantly in chloroplasts, mitochondria, and peroxisomes. In chloroplasts, salinity-induced stomatal closure reduces CO_2_ availability, leading to the over-reduction of the photosynthetic electron transport chain (ETC) (Li L et al., 2022). This forces electron leakage to molecular oxygen (O_2_) at photosystem I (PSI) via the Mehler reaction, and to a lesser extent at PSII, directly forming the superoxide radical (O_2_^−^) (Che et al., 2023; Kesawat et al., 2023). Additionally, Na^+^ accumulation impairs Rubisco, exacerbating electron leakage (Li X et al., 2022). In mitochondria, elevated Na^+^ disrupts the respiratory ETC and membrane potential, causing substantial electron leakage primarily at complexes I and III, which also yields O_2_^−^ (Zahra et al., 2022). Concurrently, increased photorespiration in peroxisomes generates primary hydrogen peroxide (H_2_O_2_) via glycolate oxidase (Sandalio et al., 2021).

Following primary generation, downstream amplification produces highly destructive ROS species. O_2_^−^ is dismutated into H_2_O_2_. Through Fenton and Haber-Weiss reactions, H_2_O_2_ interacts with transition metals to form the hydroxyl radical (OH), a highly toxic species not to be confused with the non-radical hydroxide anion (OH^−^). Lacking enzymatic scavenging mechanisms, this OH amplification is the actual biochemical origin of severe oxidative damage (Hasanuzzaman et al., 2021). The extent of these initial ROS dynamics is modulated by stress intensity, environmental factors, and species-specific traits (Sogoni et al., 2021; Ondrasek et al., 2022; Nikpour Rashidabad et al., 2025).

### 2.2 Cellular and molecular damage caused by oxidative stress

The downstream amplification of ROS, particularly the accumulation of OH and singlet oxygen (1O_2_), inflicts severe, overlapping damages across multiple cellular compartments (ElSayed et al., 2021; Fujita and Hasanuzzaman, 2022). At the cellular level, these reactive radicals target polyunsaturated fatty acids, initiating lipid peroxidation and generating toxic byproducts like MDA (Sultana et al., 2022). This directly compromises membrane integrity and disrupts ion channels vital for Na+K homeostasis (Hameed et al., 2021; Banik and Dutta, 2023). Because chloroplasts and mitochondria are primary sites of ROS production, they suffer immediate structural disorganization, including thylakoid damage and cristae swelling, which exacerbates energy deficits (Liu et al., 2021; Singh, 2022). Molecularly, oxidative stress causes protein carbonylation, inactivating key enzymes such as GAPDH and Rubisco (Fan et al., 2024; Yu et al., 2025). Furthermore, it induces DNA strand breaks and impairs repair pathways (Tomar et al., 2021; Tian et al., 2022), while altering redox-sensitive transcription factors like WRKY and MYB (Wang et al., 2021; Guo et al., 2022). If oxidative damage remains unchecked, it triggers programmed cell death in vulnerable tissues (Ebeed and El-Helely, 2021; Sarkar and Sadhukhan, 2023).

### 2.3 Antioxidant defense mechanisms in plants under salt stress

Plants deploy a combination of enzymatic and non-enzymatic defenses to counteract primary ROS generation. Enzymatic defenses include SOD, which dismutates O_2_^−^ into H_2_O_2_, followed by CAT and APX, which decompose H_2_O_2_ into water (Ahmad, 2012). The ascorbate-glutathione cycle, supported by MDHAR and glutathione reductase, maintains essential redox balance, while POD and glutathione S-transferases detoxify lipid peroxides (Gupta et al., 2015). Non-enzymatic scavengers, including ascorbate, glutathione, phenolic compounds, and tocopherols, quench primary ROS and stabilize cellular structures (Mittler, 2002). While halophytes possess robust constitutive defenses, glycophytes rely on inducible systems modulated by ABA and stress transcription factors (NAC, DREB) (Bouaziz et al., 2015; Soundararajan et al., 2019; Hussain et al., 2021).

Despite these adaptive mechanisms, severe or prolonged salt stress disrupts cellular redox homeostasis, causing ROS overproduction that easily overwhelms basal antioxidant capacities (Angon et al., 2022). This biological limitation necessitates external interventions (Kim et al., 2017). Because conventional bulk silicon often exhibits low solubility and limited cellular penetration, nanosilicon emerges as a critical and superior mitigation strategy. Unlike bulk silicon, nanosilicon efficiently penetrates cells to act as a potent signaling modulator. It directly targets and significantly upregulates the exhausted enzymatic and non-enzymatic defense systems, halting downstream ROS amplification and restoring redox homeostasis to ensure plant survival under severe salinity (Zia-ur-Rehman et al., 2023).

## 3 Nanosilicon: types, characteristics, properties, and role in oxidative stress amelioration

Nanosilicon has emerged as a promising agent for ameliorating oxidative stress in plants under salt stress conditions, owing to the unique physicochemical properties that distinguish it from bulk silicon (**Table 1**). Nanosilicon encompasses a range of silicon-based nanomaterials, primarily silicon nanoparticles (SiNPs) or silica nanoparticles (SiO_2_ NPs), engineered at the nanoscale (1–100 nm) to exploit quantum effects and high reactivity for biological applications (Fan et al., 2022; Grewal et al., 2024). Unlike bulk silicon, nanosilicon’s reduced particle size imparts unique physicochemical characteristics, such as increased surface area-to-volume ratio, enhanced solubility, and tunable surface chemistry, which facilitate efficient uptake, translocation, and interaction within plant systems (Sarkar et al., 2022). These attributes make nanosilicon a versatile tool in agriculture, particularly for mitigating abiotic stresses like salinity by modulating oxidative stress responses.

**Table 1:** Physicochemical properties of nanosilicon and their role in oxidative stress amelioration.

Property	Description	Role in oxidative stress amelioration	Reference
Particle Size	1–100 nm, nanoscale dimensions	Enhances reactivity and enables penetration into plant cell walls and membranes, targeting ROS sites	Fan et al., 2022; Wu and Li, 2022
Surface Area-to-Volume Ratio	High due to small size	Increases interaction with biological systems, adsorbs and neutralizes ROS (e.g., O_2_^-^, H_2_O_2_)	Grewal et al., 2024
Surface Chemistry	Hydroxyl (OH) groups, often functionalizable, hydrophilic	Scavenges free radicals via hydrogen donation or electron transfer	Mathur and Roy, 2020
Porosity	Mesoporous structure (2–50 nm pores, FAC pores)	Acts as a reservoir for water, nutrients, or antioxidants, supporting cellular homeostasis	Garza-Alonso et al., 2024
Crystallinity	Amorphous to crystalline, varies by synthesis method	Affects stability and solubility; amorphous forms are more biodegradable and reactive	Goswami et al., 2022; Okeke et al., 2023
Surface Charge	Typically negative due to silanol groups	Facilitates efficient uptake and distribution within plant tissues	Wu and Li, 2022
Biocompatibility/Toxicity	Low toxicity, similar to natural silicates	Ensures minimal adverse effects on plant physiology	Naidu et al., 2023
Mechanism of Action	Enhances antioxidant enzyme activity, stabilizes cellular structures, and reduces ROS	Modulates oxidative stress, improving plant tolerance to saline environments	Abdel-Haliem et al., 2017; Parveen et al., 2022

The surface of nanosilicon often contains hydroxyl (OH) groups, contributing to its hydrophilicity and ability to scavenge free radicals through hydrogen donation or electron transfer. Additionally, nanosilicon often forms mesoporous structures with pore sizes of 2–50 nm, allowing it to act as a reservoir for water, nutrients, or antioxidant molecules, releasing them under stress conditions to support cellular homeostasis (Mathur and Roy, 2020; Garza-Alonso et al., 2024). Its crystallinity, which can vary from amorphous to crystalline forms depending on synthesis methods (e.g., derived from rice husks or tetraethyl orthosilicate), influences its stability and solubility, with amorphous nanosilicon showing higher biodegradability and reactivity in planta (Goswami et al., 2022; Okeke et al., 2023). The nanoparticles’ ability to penetrate plant cell walls and membranes, attributed to their nanoscale size and surface charge (typically negative due to silanol groups), allows for efficient uptake and distribution within tissues, targeting sites of ROS production like chloroplasts and mitochondria (Newkirk et al., 2021; Wu and Li, 2022). Furthermore, nanosilicon’s low toxicity and biocompatibility, stemming from its similarity to naturally occurring silicates, ensure minimal adverse effects on plant physiology, unlike some metal-based nanoparticles (Naidu et al., 2023). These physicochemical traits collectively enable nanosilicon to modulate oxidative stress by enhancing antioxidant enzyme activity, stabilizing cellular structures, and reducing ROS accumulation, positioning it as a superior alternative to bulk silicon for stress mitigation in agriculture under most tested conditions (Abdel-Haliem et al., 2017; Parveen et al., 2022).

Nanosilicon can be classified into several types based on structure, composition, and synthesis methods, each with distinct characteristics that influence their behavior in planta:

Amorphous Silica Nanoparticles: Amorphous silica nanoparticles (a-SiO_2_ NPs) represent the non-crystalline form of silica, typically produced through chemical routes such as sol–gel synthesis or via biological extraction from agricultural residues. These nanoparticles possess a disordered atomic arrangement and contain abundant surface silanol (Si–OH) groups, which impart a net negative surface charge under neutral to slightly alkaline conditions. Compared with crystalline silica, a-SiO_2_ exhibits higher reactivity and a moderate but appreciably greater dissolution rate, gradually converting to plant-available monosilicic acid (Si(OH)_4_). This dissolution behavior reduces their long-term persistence in soils (Chittick et al., 1969; Mangolini, 2013; Zexer et al., 2023).

Crystalline Silicon Nanoparticles: Crystalline silicon nanoparticles consist of highly ordered silicon lattices and are typically produced through physical techniques such as laser ablation or chemical vapor deposition. Owing to their well-defined crystalline structure, these nanoparticles exhibit high structural stability and extremely low solubility compared with amorphous forms. Their sizes commonly fall within the 5–20 nm range, where quantum confinement effects become significant, leading to enhanced optical and electronic properties such as improved photoluminescence and charge-carrier behavior (Mangolini, 2013).

Mesoporous Silica Nanoparticles: Mesoporous silica nanoparticles (MSNs) possess highly ordered pore networks with mesopore diameters typically ranging from 2 to 50 nm, providing an exceptional internal surface area and high loading capacity for nutrients, agrochemicals, or antioxidant molecules. They are commonly synthesized through template-assisted routes using surfactant structure-directing agents, enabling precise control over pore architecture and particle morphology. MSNs also offer tunable surface functional groups that facilitate targeted delivery and controlled release. Their particle sizes generally fall within the 50–100 nm range, with high porosity that may reach up to 50% internal void volume (Sun et al., 2016; Ghaferi et al., 2021).

Functionalized or Composite Nanosilicon: These involve surface modifications (e.g., with polymers, metals, or organic ligands) to enhance specificity, such as PEG-coated SiO_2_ NPs for improved biocompatibility. Characteristics include altered zeta potential (surface charge) and hydrophilicity, which affect cellular uptake and stability in varying pH environments (Li et al., 2021). However, it is important to note that while these functionalized composites show immense potential, comprehensive in planta experimental evidence regarding their efficacy and long-term behavior in agricultural systems remains limited.

In agricultural contexts, the predominant types are a-SiO_2_ NPs and MSNs, owing to their cost-effectiveness, scalability, and efficacy in enhancing plant resilience (Rastogi et al., 2019; Goswami et al., 2022). Amorphous SiO_2_ NPs are the most widely applied, particularly for abiotic stress mitigation, as they promote nutrient uptake, upregulate antioxidant enzymes, and reduce ROS accumulation by 10–50% in different crops (Farhangi-Abriz and Torabian, 2018; Wang et al., 2022). Their high solubility ensures rapid bioavailability, but efficacy depends on particle size; smaller particles (<20 nm) penetrate roots and leaves more effectively, though they risk aggregation in high-salinity soils (Rastogi et al., 2019).

MSNs excel in controlled-release applications, such as delivering micronutrients or phytohormones, which synergize with stress tolerance mechanisms. For instance, MSNs loaded with abscisic acid have shown prolonged release, enhancing osmotic adjustment and reducing oxidative damage in salt-stressed plants (Elmenofy et al., 2025). Crystalline Si NPs are less common in agriculture due to higher production costs and potential phototoxicity under UV exposure, but they hold promise for precision farming via sensor integration (Rastogi et al., 2019).

Analyzing these types, amorphous silica nanoparticles offer broad-spectrum benefits for oxidative stress alleviation at low doses, outperforming bulk silicon in penetration and reactivity, but may induce phytotoxicity above optimal thresholds. MSNs provide superior tunability for integrated pest and stress management, minimizing environmental runoff, though their complexity increases synthesis costs (Rastogi et al., 2019). Functionalized variants address limitations like poor dispersibility, enhancing targeted delivery in saline environments.

While the ameliorative effects of nanosilicon are widely documented, it is critical to distinguish between experimentally validated outcomes and proposed mechanistic pathways regarding ROS scavenging. Numerous in planta studies consistently validate that nanosilicon application significantly upregulates the activity of key endogenous antioxidant enzymes (such as SOD, CAT, and APX), reduces oxidative damage markers like MDA and H_2_O_2_, and modulates stress-responsive genes within the ABA and MAPK signaling cascades. In contrast, the direct physical scavenging of ROS via surface reactive silanol (Si-OH) groups remains a largely proposed pathway *in vivo*. Although *in vitro* chemical assays support the radical-scavenging capacity of Si-OH groups, the extent of this direct, stoichiometric interaction within the complex cellular milieu is still debated. Therefore, it is currently hypothesized that while direct Si-OH scavenging may offer localized and immediate ROS quenching upon cellular entry, the sustained and primary mechanism for oxidative stress mitigation is predominantly driven by the nanosilicon-induced, signaling-mediated amplification of the plant’s endogenous defense machinery.

## 4 Interaction with plant cells and ROS

Nanosilicon interacts intricately with plant cells and ROS under salt stress, leveraging its nanoscale properties to mitigate oxidative damage and bolster cellular resilience. Due to its small size (1-100 nm) and high specific surface area, nanosilicon can penetrate plant cell walls, which typically possess porous structures with openings of 5-20 nm. It is widely proposed that traversal across plasma membranes occurs via endocytosis, diffusion, or transporter-mediated uptake; however, while the cellular internalization of nanosilicon is experimentally confirmed, the exact proportional contribution of these specific uptake routes remains largely hypothetical and requires further in planta validation.

Once inside the cell, it is commonly hypothesized that nanosilicon targets and accumulates in ROS-rich organelles such as the cytoplasm, chloroplasts, and mitochondria (Farhangi-Abriz and Torabian, 2018; Dhiman et al., 2021). A critical distinction must be made here: while the functional protection of these organelles is well-documented, direct ultrastructural evidence of organelle-specific nanosilicon accumulation is still limited. Current models infer this intracellular targeting primarily from biochemical outcomes rather than direct visualization. Within these compartments, a proposed mechanistic pathway suggests that nanosilicon directly interacts with ROS, including superoxide, hydrogen peroxide, and hydroxyl radicals, through its surface reactive Si−OH) groups. These groups can hypothetically donate hydrogen or accept electrons to neutralize unstable molecules (Sulaiman et al., 2023; Wahi et al., 2024), thereby preventing lipid peroxidation of membranes, protein oxidation, and DNA damage (Akyol et al., 2020).

In contrast to the hypothesized direct physical scavenging and specific organelle accumulation, the indirect biochemical reinforcement provided by nanosilicon constitutes well-established evidence. Extensive experimental data demonstrate that nanosilicon robustly enhances the plant’s endogenous antioxidant defenses by upregulating the activity of enzymes like SOD, CAT, and APX, potentially modulated through signaling pathways involving jasmonic acid or salicylic acid (Farhangi-Abriz and Torabian, 2018; Shivappa et al., 2026). Furthermore, it is proven to stabilize cell walls through silica deposition, significantly reducing membrane leakage and maintaining ion homeostasis under salinity-induced stress (Avestan et al., 2019; Dhiman et al., 2021).

In chloroplasts, functional assays provide strong evidence that nanosilicon mitigates photooxidative stress by protecting photosystem II (PSII) from ROS-induced damage and facilitating electron transport, thus limiting further ROS generation (Xiong et al., 2024). Concrete experimental evidence from crops such as faba bean, triticale, and tomato clearly shows that nanosilicon application decreases H_2_O_2_ and MDA levels while increasing chlorophyll content, unambiguously underscoring its protective role (Qados, 2015; Aghaei et al., 2024; Wang et al., 2025). However, the magnitude of these interactions is highly dependent on variables such as nanosilicon concentration, particle size, and plant species, with optimal doses enhancing ROS detoxification without causing cytotoxicity. By bridging the proposed physical ROS neutralization with well-established biochemical reinforcement, nanosilicon provides a multifaceted strategy to combat oxidative stress, making it a vital tool for enhancing salt tolerance (Grewal et al., 2024).

## 5 Advantages of nanosilicon over conventional silicon

Nanosilicon offers significant advantages over conventional silicon in mitigating oxidative stress and enhancing plant tolerance to salt stress, primarily due to its nanoscale-specific properties (**Table 2**) that amplify its efficacy and bioavailability (Cao and Wang, 2022). Unlike conventional silicon, typically applied as silicates or bulk forms like sodium silicate, nanosilicon’s particle size (1–100 nm) provides a vastly increased surface area-to-volume ratio, increasing reactivity with plant cells and ROS (Mathur and Roy, 2020). This enables efficient penetration into cell walls and membranes, reaching intracellular sites of ROS productio, such as chloroplasts and mitochondria, locations less accessible to bulk silicon due to its larger particle size and lower solubility (Alam et al., 2022). Enhanced solubility and dispersibility of nanosilicon in aqueous environments further improve its delivery and absorption, ensuring a more uniform distribution within plant tissues compared to uneven deposition often with bulk silicon (Ismail et al., 2022). Functionally, nanosilicon’s reactive silanol groups enable direct scavenging of O_2_^-^ and H_2_O_2_, a capability less pronounced in conventional bulk silicon due to its lower surface reactivity (Dhiman et al., 2021). Nanosilicon also more effectively upregulates antioxidant enzyme activity (e.g., SOD, CAT, APX) and promotes silica deposition in cell walls, strengthening structural integrity and reducing ion toxicity under salt stress (Fan et al., 2022). Comparative studies have demonstrated that nanosilicon application results in greater reductions in oxidative damage markers (e.g., MDA levels) and higher photosynthetic rates than equivalent doses of conventional silicon, highlighting its superior protective capacity (Riaz et al., 2022; Shoukat et al., 2024 B). Moreover, its efficacy at lower application rates makes it a cost-effective and environmentally friendly alternative, minimizing resource use and potential soil accumulation (Kheyri, 2022). However, while conventional silicon is less expensive and more widely available, nanosilicon’s tunable synthesis, such as porosity and surface charge, provides a precision that aligns better with the dynamic needs of plants under stress, positioning it as a next-generation solution for sustainable agriculture in saline environments (Mathur and Roy, 2020; Grewal et al., 2024). It is important to note, however, that comparative data between nanosilicon and bulk silicon are still relatively sparse and heavily rely on a limited set of crops, such as wheat, rice, and maize. Therefore, the superiority of nanosilicon may be species-dependent, and further studies across diverse plant taxa are required to confirm its universal applicability.

**Table 2:** Advantages of nanosilicon over conventional silicon.

Aspect	Nanosilicon characteristics	Conventional silicon characteristics	Advantage of nanosilicon	Reference
Particle Size and Surface Area	1–100 nm, high surface area-to-volume ratio	Larger particles, lower surface area	Greater reactivity, better cellular interaction	Mathur and Roy, 2020; Hajihashemi and Kazemi, 2022
Cellular Penetration	Efficiently penetrates cell walls and membranes, targets chloroplasts and mitochondria	Limited uptake due to larger size, low solubility	Reaches ROS production sites effectively	Farouk, 2022
Solubility and Dispersibility	High solubility, uniform tissue distribution	Lower solubility, uneven deposition	Improved delivery and absorption in plants	Ismail et al., 2022
ROS Scavenging	Reactive silanol (Si-OH) groups scavenge O_2_^-^, H_2_O_2_	Lower surface reactivity, limited ROS scavenging	More effective ROS neutralization	Dhiman et al., 2021
Antioxidant Enzyme Upregulation	Strongly upregulates SOD, CAT, APX	Slower, less pronounced enzyme activation	Enhances antioxidant defenses efficiently	Fan et al., 2022
Cell Wall Reinforcement	Promotes silica deposition, reduces ion toxicity	Slower silica deposition, less structural support	Strengthens cell walls, improves ion homeostasis	Fan et al., 2022
Protective Capacity	Greater reduction in MDA, higher photosynthetic rates	Less reduction in oxidative damage, lower photosynthetic efficiency	Superior protection against oxidative stress	Ahmed et al., 2024; Hajizadeh et al., 2021
Application Efficiency	Lower doses needed, cost-effective, less soil accumulation	Higher doses required, potential soil buildup	Environmentally friendly, resource-efficient	Noctor et al., 2012
Synthesis and Tunability	Tailored synthesis, tunable porosity, surface charge	Simpler production, less tunable	Precise, adaptable to plant stress needs	Grewal et al., 2024; Mathur and Roy, 2020

**Figure 1:**
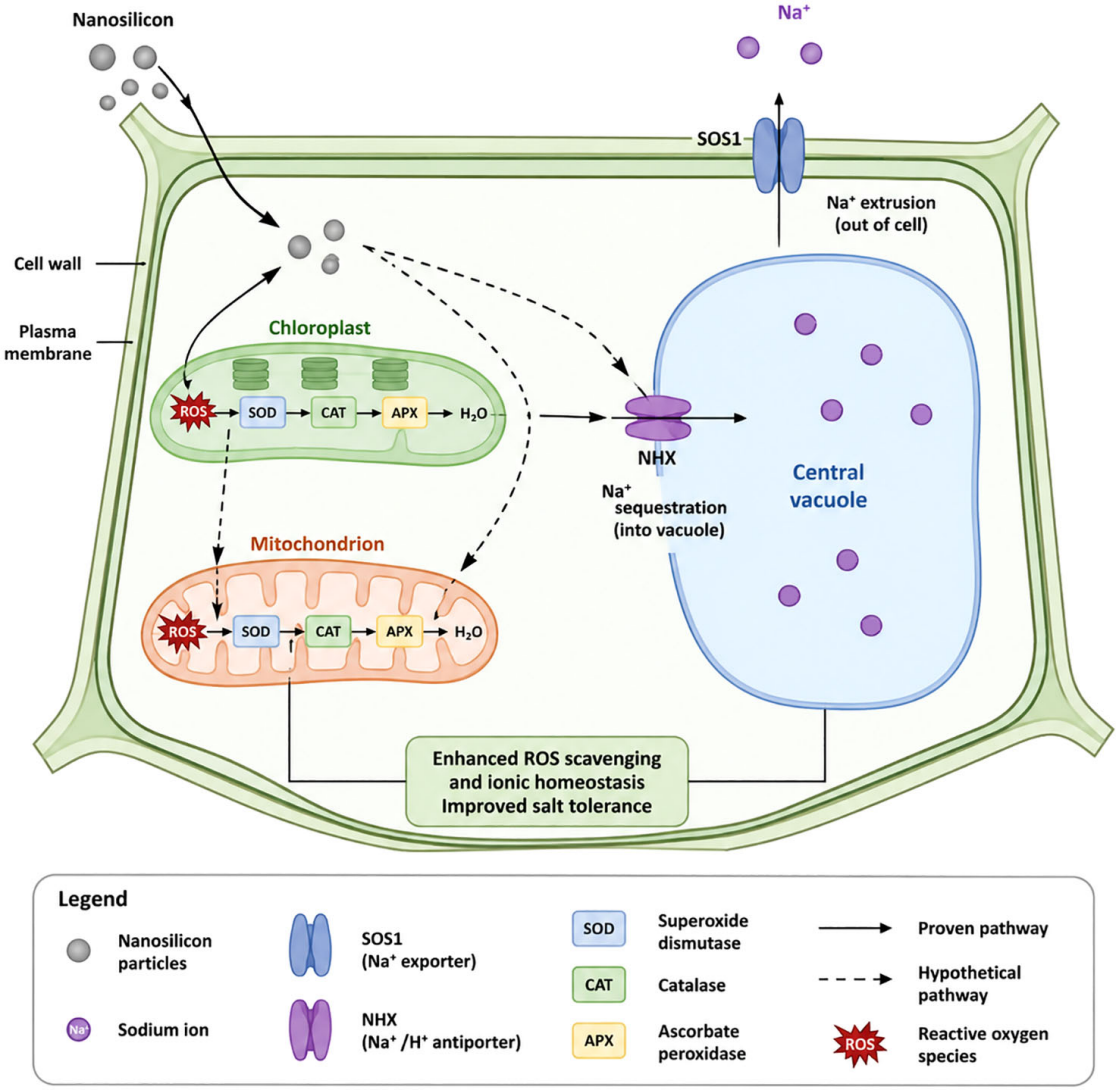
Schematic representation of the cellular mechanisms induced by nanosilicon (SiNPs) to mitigate salinity stress in plant cells. The diagram illustrates the dual regulatory role of SiNPs in maintaining ionic homeostasis and enhancing ROS scavenging. To prevent sodium toxicity, SiNPs promote Na^+^ extrusion from the cytosol into the apoplast via the plasma membrane SOS1 exporter and facilitate Na^+^ sequestration into the central vacuole through the tonoplast NHX antiporter. Concurrently, SiNPs alleviate oxidative stress by stimulating the antioxidant defense system, including the enzymatic cascade of SOD, CAT, and APX, in chloroplasts and mitochondria, thereby detoxifying reactive oxygen species (ROS) into H_2_O. Solid arrows represent well-established biochemical pathways, whereas dashed arrows indicate hypothetical regulatory signaling pathways modulated by SiNPs, ultimately contributing to improved salt tolerance.

The physiological improvements induced by nanosilicon under salinity stress are driven by a complex, tripartite mechanism: acting as a physical barrier, a signaling modulator, and through direct biochemical interactions. First, as a physical barrier, nSi integrates deeply into the cellulose-hemicellulose network of the cell wall, reinforcing rigidity and restricting the bypass flow of toxic Na^+^ and Cl^−^ ions. Second, acting as a potent signaling modulator due to its enhanced cellular penetration, nSi acts as an elicitor that amplifies phytohormone signaling (particularly ABA for rapid stomatal regulation) and triggers MAPK cascades to upregulate stress-responsive and antioxidant genes. Finally, through direct biochemical interactions facilitated by its high surface area-to-volume ratio, nSi stabilizes membrane lipid bilayers against salt-induced phase transitions and interacts with the functional groups of antioxidant enzymes (such as SOD and CAT) to enhance their catalytic efficiency. Consequently, while providing essential physical fortification, nSi’s superior efficacy primarily stems from its dynamic roles in intracellular signaling and biochemical interactions. To elucidate the protective role of silicon nanoparticles against salinity-induced cellular damage, a comprehensive mechanistic model is proposed (**Figure 1**). Under salt stress conditions, excessive accumulation of Na^+^ disrupts cellular function and induces severe oxidative stress. SiNPs mitigate this toxicity primarily through two interconnected pathways: maintenance of ionic homeostasis and enhancement of the antioxidant defense system. As illustrated, SiNPs regulate intracellular Na^+^ levels by stimulating the activity of the plasma membrane SOS1 exporter, which extrudes Na^+^ from the cytosol into the apoplast, and by upregulating the tonoplast NHX antiporter, which sequesters Na^+^ into the central vacuole. Furthermore, SiNP application alleviates oxidative stress by modulating ROS-scavenging systems within vital energy-producing organelles, namely chloroplasts and mitochondria. This response involves a sequential enzymatic cascade comprising SOD, CAT, and APX, which effectively detoxifies ROS into water. The synergy between established and proposed SiNP-mediated pathways coordinates ionic balance and robust ROS detoxification, ultimately leading to improved salt tolerance in plants.

## 6 Mechanisms of nanosilicon in alleviating oxidative stress

### 6.1 Enhancement of enzymatic antioxidants (e.g., SOD, CAT, APX)

Nanosilicon alleviates oxidative stress in plants under salt stress through multiple complementary mechanisms, exploiting its unique nanoscale properties to modulate cellular metabolism and neutralize the damaging effects of ROS (Singh et al., 2024). By penetrating plant tissues and influencing biochemical pathways, nanosilicon enhances the plant’s capacity to cope with salinity-induced oxidative damage, offering a robust defense against ROS accumulation and its downstream consequences (Fan et al., 2022; Grewal et al., 2024). One of the primary mechanisms by which nanosilicon mitigates oxidative stress is through the significant enhancement of enzymatic antioxidants, including SOD, CAT, and APX, which collectively form a critical line of defense against ROS in plants (Farhangi-Abriz and Torabian, 2018; Fan et al., 2022; Riaz et al., 2022). SOD, the first step in ROS detoxification, catalyzes the dismutation of superoxide radicals into hydrogen peroxide. It is hypothesized that nanosilicon might stabilize SOD structure, potentially through direct interaction with its active sites; however, direct structural biochemistry evidence is still required to confirm this mechanism (Singh et al., 2023; Nongbet et al., 2024). Subsequently, CAT, predominantly located in peroxisomes, decomposes H_2_O_2_ into water and oxygen; nanosilicon enhances its catalytic efficiency, as evidenced by higher CAT activity observed in wheat and pea under salinity compared to untreated controls (Hajihashemi and Kazemi, 2022; Ismail et al., 2022). APX, a key component of the ascorbate-glutathione cycle, further reduces H_2_O_2_ using ascorbate as a substrate, and nanosilicon boosts its activity by elevating ascorbate levels and upregulating APX gene expression in chloroplasts and cytosol, where ROS production peaks under salinity (Qados, 2015). These effects are likely mediated by improved cellular redox status, either by directly scavenging ROS to reduce oxidative pressure or by triggering signaling cascades that involve transcription factors like WRKY and MYB, which regulate antioxidant genes (Fan et al., 2022; Farouk, 2022; Ahmed et al., 2024). Experimental studies, such as those on pea and triticale, demonstrated that nanosilicon application under salt stress increased SOD, CAT, and APX activities by 20–50% and correlated with reduced H_2_O_2_ levels and lipid peroxidation (Qados, 2015; Ismail et al., 2022; Aghaei et al., 2024). The small size and high surface reactivity of nanosilicon enable it to penetrate cellular compartments, delivering localized support to these enzymes. This coordinated upregulation of enzymatic antioxidants not only curtails oxidative damage but also sustains metabolic functions like photosynthesis and ion homeostasis, underscoring nanosilicon’s pivotal role in bolstering plant resilience to salt-induced oxidative stress (Ismail et al., 2022; Riaz et al., 2022; Sayed et al., 2022).

### 6.2 Boosting non-enzymatic antioxidant systems

In addition to enzyme activation, nanosilicon enhances non-enzymatic antioxidant systems, particularly glutathione (GSH) and ascorbate, providing an additional layer of protection against ROS (Dhiman et al., 2021; Hajizadeh et al., 2021). GSH, a tripeptide (γ-glutamyl-cysteinyl-glycine), serves as a redox buffer and regenerates other antioxidants like ascorbate through the ascorbate-glutathione cycle (Noctor et al., 2012; Hasanuzzaman et al., 2017; Koh et al., 2021). Nanosilicon boosts GSH levels by upregulating the activity of biosynthetic enzymes like γ-glutamylcysteine synthetase (γ-ECS) and glutathione synthetase (GS), as well as glutathione reductase (GR), which recycles oxidized glutathione (GSSG) to its reduced form, maintaining a high GSH/GSSG ratio critical for cellular redox homeostasis (Riaz et al., 2022). Similarly, ascorbate (vitamin C), a potent water-soluble antioxidant, quenches ROS such as O_2_^-^ and singlet oxygen (¹O_2_), protecting membranes and proteins from oxidative damage (Hameed et al., 2015; Shalata and Neumann, 2021). Nanosilicon increases ascorbate biosynthesis via the L-galactose pathway and promotes its regeneration through monodehydroascorbate reductase and dehydroascorbate reductase, both dependent on GSH as a cofactor (Farhangi-Abriz and Torabian, 2018; Sayed et al., 2022). By reducing oxidative load, nanosilicon preserves antioxidant pools and stabilizes redox metabolisms (Farhangi-Abriz and Torabian, 2018). Studies on salt-stressed plants, including faba bean, wheat, and tomato, demonstrate that the application of nanosilica increases GSH and ascorbate concentrations by 30% to 60%, a response closely associated with reduced ROS levels and lipid peroxidation (Qados, 2015; Riaz et al., 2022). A similar protective effect has also been observed under other abiotic stresses, such as cadmium toxicity in tomato (Rahmatizadeh et al., 2024). Furthermore, nanosilicon’s high surface reactivity ensures to interact directly with these molecules, enhancing their stability or availability at sites of oxidative stress, such as the apoplast and chloroplasts, resulting in faster and stronger responses than conventional silicon (Fan et al., 2022; Verma et al., 2022). This reinforcement not only detoxifies ROS but also supports enzymatic antioxidants and protects cellular integrity, making nanosilicon a vital enhancer of non-enzymatic defenses in salt-stressed plants (Verma et al., 2022).

### 6.3 Reduction of ROS accumulation and lipid peroxidation

Nanosilicon plays a critical role in reducing the accumulation of ROS and mitigating lipid peroxidation, two interconnected processes that exacerbate oxidative stress in plants under salt stress (Farhangi-Abriz and Torabian, 2018; Hajihashemi and Kazemi, 2022). Its reactive silanol (Si-OH) groups donate hydrogen or accept electrons, neutralizing ROS, including (O_2_^-^), H_2_O_2_, and OH^-^, before they damage cellular components. This direct ROS-quenching ability is complemented by nanosilicon’s enhancement of both enzymatic and non-enzymatic antioxidant systems, which collectively lower ROS levels by improving detoxification efficiency; SOD converts O_2_^-^ to H_2_O_2_, which CAT and APX then degrade, while glutathione and ascorbate mop up residual radicals (Fan et al., 2022; Riaz et al., 2022). Experimental evidence from salt-stressed plants like tomato and triticale treated with nanosilicon showed up to 40% lower H_2_O_2_ and significantly reduced lipid peroxidation, as evidenced by decreases in MDA and HNE by as much as 50% (Sayed et al., 2022; Aghaei et al., 2024). Lower peroxidation helps maintain membrane compartmentalization and ion homeostasis (e.g., limiting Na^+^ influx) (Fan et al., 2022; Hajihashemi and Kazemi, 2022). This protective effect is partly due to nanosilicon’s deposition as silica in cell walls, which strengthens structural barriers and reduces ROS diffusion into sensitive areas, and partly due to its modulation of lipid metabolism, potentially upregulating genes involved in membrane repair or fatty acid desaturation (Sarkar et al., 2022; Kaur et al., 2023). Compared with bulk silicon, nanosilicon’s smaller size and greater reactivity enable rapid targeting of oxidative damage sites, resulting in more effective and sustained protection (Etesami et al., 2022; Grewal et al., 2024). In general, nanosilicon can directly alleviate oxidative stress by enhancing the activity of antioxidant enzymes, promoting the synthesis of non-enzymatic antioxidants, reducing ROS and lipid peroxidation, and sustaining photosynthetic performance and overall plant growth.

### 6.4 Regulation of oxidative stress-related gene expression

Nanosilicon alleviates oxidative stress in plants under salt stress by modulating the expression of genes associated with oxidative stress responses, thereby enhancing adaptive capacity at the molecular level (Almutairi, 2016; Zhang et al., 2023). Transcriptomic analyses consistently demonstrate that nanosilicon application results in the upregulation of genes encoding key antioxidant enzymes, such as Cu/Zn-SOD, Mn-SOD, CAT1, and APX1, which are critical for detoxifying O_2_^−^ and H_2_O_2_. In salt-stressed crops, nanosilicon application increases their transcript levels by 1.5 to 3-fold compared with untreated plants (Roychoudhury, 2023; Singh et al., 2023). It is crucial to note that this gene-level regulation is generally inferred to be an indirect effect. The enhancement is likely mediated through nanosilicon’s modulation of upstream stress signaling pathways, such as those involving ROS as secondary messengers and phytohormones like abscisic acid and salicylic acid, rather than direct physical binding of the nanoparticles to DNA. These pathways subsequently activate transcription factors like WRKY, MYB, and NAC, which bind to promoter regions of antioxidant genes and amplify their expression (Farouk, 2022; Nicolau et al., 2024). Additionally, transcript levels of genes linked to non-enzymatic antioxidants, such as γ\gammaγ-ECS and GS for glutathione biosynthesis and GalLDH for ascorbate production, are elevated following nanosilicon treatment. It also downregulates the expression of genes associated with ROS generation, such as RBOH (respiratory burst oxidase homolog) (Al-Khayri et al., 2023). For instance, rice under salt stress showed suppressed RBOH expression alongside elevated SOD and CAT transcripts, resulting in a balanced redox state.

While this regulatory outcome is facilitated by nanosilicon’s ability to penetrate cells, hypotheses suggesting its direct interaction with nuclear components or direct physical triggering of signaling cascades remain proposed models requiring further *in planta* elucidation (Almutairi, 2019; Zhou et al., 2021). Furthermore, while it has been hypothesized that nanosilicon might stabilize mRNA transcripts or influence epigenetic modifications like histone acetylation, direct experimental evidence supporting nanosilicon-specific epigenetic effects is currently lacking and remains an area for future validation. By fine-tuning this genetic network indirectly, nanosilicon suppresses ROS accumulation and enhances cellular resilience, offering a molecular basis for its superiority over bulk silicon in mitigating salt-induced oxidative stress (Almutairi, 2016; Dhiman et al., 2021; Sharma et al., 2023; Singh et al., 2024). The main mechanisms by which nanosilicon regulates oxidative stress in plants under salt stress are summarized in **Table 3**.

**Table 3:** Comprehensive biochemical and molecular mechanisms of nanosilicon in alleviating oxidative stress.

Category/mechanism	Description	Role in oxidative stress mitigation	Reference
Biochemical & Physiological Enhancement of Enzymatic Antioxidants	Upregulates SOD, CAT, APX via gene expression (e.g., Cu/Zn-SOD, CAT1, APX1) and stabilizes enzyme structure	Detoxifies O_2_^-^ and H_2_O_2_, increases enzyme activity by 20–50%, reduces oxidative damage	Farhangi-Abriz and Torabian, 2018; Ismail et al., 2022; Fan et al., 2022; Wang et al., 2023
Redox Homeostasis & Non-Enzymatic Antioxidants	Elevates GSH and ascorbate (30–60%) via upregulation of γ-ECS, GS, and regeneration enzymes (GR, MDHAR, DHAR)	Neutralizes ROS, maintains GSH/GSSG ratios, protects membranes, stabilizes chloroplast/mitochondrial function	Dhiman et al., 2021; Sayed et al., 2022; Qados, 2015; Ahmed et al., 2024; Laifa et al., 2024; Seyed Hajizadeh et al., 2023
ROS Reduction & Lipid Peroxidation	Directly scavenges ROS via silanol groups, reduces H_2_O_2_ by 25–40%, lowers MDA by 30–50%	Minimizes ROS damage, stabilizes membranes, and maintains ion homeostasis	Sayed et al., 2022; Fan et al., 2022; Kaur et al., 2025; Rahmatizadeh et al., 2024
Synergy with Osmotic/Ion Tolerance	Promotes proline (40%), lowers Na^+^/K^+^ ratio (25%), increases chlorophyll (20–35%), upregulates LEA, DREB	Enhances osmotic adjustment, ion homeostasis, photosynthesis, and stress gene expression	Sarkar et al., 2022; Grewal et al., 2024; Rahmatizadeh et al., 2024; Bhat et al., 2021
Signaling Pathways & Gene Regulation	Modulates ABA, SA, JA, and MAPK pathways; upregulates WRKY, MYB, NAC; downregulates RBOH	Enhances antioxidant gene expression (1.5–3-fold), reduces ROS production, boosts stress tolerance	Hasanuzzaman et al., 2017; Verma et al., 2022; Sharma et al., 2023; Mostofa et al., 2021; Alharbi et al., 2022; Lemos Neto et al., 2021

## 7 Molecular and biochemical insights

### 7.1 Nanosilicon’s role in signaling pathways linked to oxidative stress

Nanosilicon’s protective role in salt stress extends beyond direct ROS scavenging, encompassing profound molecular and biochemical influences that reshape cellular responses (Singh et al., 2024). By interacting with signaling pathways and modulating biochemical processes, nanosilicon functions as a regulatory agent that amplifies defense responses against salt-induced oxidative stress (Mostofa et al., 2021; Fan et al., 2022). Upon entering plant cells, nanosilicon interacts with redox-sensitive pathways, potentially functioning as a mild stressor or co-factor to trigger adaptive responses (Cameron et al., 2022). One critical mechanism involves ROS themselves as secondary messengers, where nanosilicon’s initial ROS-scavenging action reduces excessive H_2_O_2_ levels, fine-tuning its signaling role to activate transcription factors like WRKY, MYB, and NAC, which upregulate antioxidant genes such as SOD, CAT, and APX (Singh et al., 2023). In salt-stressed rice, nanosilicon doubled WRKY6 expression, which correlated with increased CAT activity and reduced oxidative damage (Wang et al., 2023). In addition to ROS-dependent signaling, nanosilicon influences phytohormone-mediated pathways, particularly those involving ABA, which orchestrates stomatal closure and stress gene expression under salinity (Eghlima et al., 2024). By stabilizing ABA signaling, possibly through interactions with protein kinases like SnRK2, nanosilicon amplifies the expression of ABA-responsive genes, such as RD29A, enhancing osmotic and oxidative stress tolerance (Ain et al., 2024). Salicylic acid and jasmonic acid pathways are also modulated, with nanosilicon boosting NPR1 (a SA regulator) and JAZ (a JA repressor) expression in rice, linking it to systemic acquired resistance and ROS detoxification (Myers et al., 2023; Wang et al., 2025). Furthermore, nanosilicon may interact with mitogen-activated protein kinase (MAPK) cascades, such as MPK3/MPK6, which relay stress signals to the nucleus (Li Z et al., 2022). This signaling enhancement reduces the activity of ROS-generating enzymes like NADPH oxidase (RBOH), curbing apoplastic ROS bursts (Mostofa et al., 2021; Singh et al., 2025). Unlike conventional silicon, nanosilicon’s nanoscale penetration enables it to target signaling hubs in chloroplasts and mitochondria more effectively. By integrating ROS scavenging with signal amplification, nanosilicon establishes a feedback loop that primes plants for sustained resilience (Fan et al., 2022; Grewal et al., 2024).

### 7.2 Interaction with redox homeostasis

Nanosilicon plays a central role in restoring redox homeostasis, the dynamic balance between ROS generation and detoxification, which is severely disrupted by salinity in organelles like chloroplasts, mitochondria, and peroxisomes (Hossain and Dietz, 2016; Basu and Kumar, 2021). Building upon the primary antioxidant defenses, nanosilicon’s intervention fundamentally prevents the cellular shift toward a pro-oxidant state by comprehensively modulating both enzymatic and non-enzymatic pools (Alharbi et al., 2022; Aghaei et al., 2024; Wang et al., 2025). Rather than merely scavenging ROS, nanosilicon critically sustains essential redox couples, specifically the GSH/GSSG and ascorbate/dehydroascorbate ratios, which regulate the cellular thiol-disulfide status and protect structural proteins from oxidation (Lemos Neto et al., 2021; Xiong et al., 2024; Zhang et al., 2024). This is supported by its ability to enhance the broader ascorbate-glutathione cycle and increase reduced GSH levels under stress (Raza et al., 2023; Kusvuran et al., 2024). At the more advanced molecular level, nanosilicon explicitly modulates redox-sensitive protein networks, such as thioredoxins and peroxiredoxins, which fine-tune metabolic processes like photosynthesis and respiration to minimize ROS leakage at the source (Liu et al., 2021). Furthermore, its nanoscale properties actively stabilize mitochondrial membranes and chloroplast thylakoid functions, limiting electron leakage and sustaining energy metabolism (Dhiman et al., 2021; Alam et al., 2022). This internal regulation is complemented by nanosilicon’s structural deposition in cell walls, which restricts Na^+^ influx and mitigates the ionic stress that typically exacerbates ROS generation (Dhiman et al., 2021). Collectively, these advanced redox-regulatory mechanisms position nanosilicon as a superior agent in maintaining cellular equilibrium compared to bulk silicon, ensuring long-term plant survival in saline environments (Akhter et al., 2022; Laifa et al., 2024).

### 7.3 Synergy with other stress tolerance mechanisms

Nanosilicon also synergizes with other stress tolerance mechanisms, integrating physiological, biochemical, and molecular strategies that collectively enhance resilience under salinity. For example, nanosilicon promotes the accumulation of compatible solutes like proline, glycine betaine, and soluble sugars, which maintain osmotic balance and serve as secondary ROS scavengers (Sayed et al., 2022; Shoukat et al., 2024b), as seen in salt-stressed wheat, where nanosilicon increased proline levels by 40% alongside a 30% reduction in H_2_O_2_ (Hajihashemi and Kazemi, 2022). This synergy extends to ion homeostasis, where nanosilicon’s deposition as silica in cell walls and its modulation of Na^+^/K^+^ transporters (e.g., HKT and NHX) reduce sodium uptake and compartmentalization, mitigating ionic stress that fuels ROS production (Dhiman et al., 2021), studies on maize show a remarkable decrease in Na^+^/K^+^ ratio with nanosilicon, correlating with lower oxidative damage (Ali et al., 2021). Furthermore, nanosilicon enhances photosynthetic protection by stabilizing chlorophyll and PSII under salinity, reducing photooxidative ROS bursts (Seyed Hajizadeh et al., 2023; Grewal et al., 2024), as evidenced by a 20–35% increase in chlorophyll content in wheat, which complements its antioxidant effects by sustaining energy supply for defense responses (Hajihashemi and Kazemi, 2022). At the molecular level, nanosilicon interacts with ABA and calcium-dependent protein kinases (CDPKs), amplifying the expression of stress-responsive genes like LEA and DREB, which preserve cellular structure and improve dehydration tolerance (Li Z et al., 2022; Sarkar et al., 2022). This cooperative action also involves crosstalk with heat shock proteins (HSPs) and aquaporins, where nanosilicon’s presence enhances HSP accumulation for protein stabilization and improves water uptake efficiency, further alleviating oxidative pressure (Khan et al., 2024; Bhardwaj et al., 2023; Khursheed et al., 2025). Unlike bulk silicon, nanosilicon’s nanoscale penetration fosters an integrated response; for instance, in barley, it simultaneously boosted SOD activity and proline synthesis, reducing MDA by 50% more than conventional silicon (Akhter et al., 2022). This synergy fortifies both antioxidant defenses and broader tolerance mechanisms, enabling plants to withstand prolonged salinity stress, making nanosilicon a uniquely versatile tool for stress mitigation in agriculture (Verma et al., 2022; Sarkar et al., 2022; Grewal et al., 2024). **Table 3** summarizes the main molecular and biochemical insights into the role of nanosilicon in alleviating oxidative stress.

Current experimental evidence confirms that nanosilicon application significantly alters the expression profiles of stress-responsive genes under salinity, enhancing the plant’s adaptive transcriptional landscape. For instance, under salinity stress, nanosilicon can upregulate ABA biosynthesis and ABA-responsive genes, promoting stomatal regulation and improved water status. However, the exact upstream signaling mechanisms linking nanosilicon perception to these downstream transcriptional changes remain largely putative. While it is well-established that salt stress activates complex signaling networks, including phytohormone cross-talk and MAPK cascades, the direct physical or biochemical interaction of nanosilicon with these initial signaling sensors has not been definitively proven *in vivo*. In fact, reported effects of nanosilicon on MAPK signaling can be highly variable, sometimes appearing suppressive rather than activating, depending on the species and study (Rios et al., 2017). Typically, generalized MAPK activation can phosphorylate downstream transcription factors, such as WRKYs and in some contexts MYB-related factors, thereby enhancing the expression of antioxidant defense genes and strengthening ROS scavenging capacity (Zhang et al., 2006).

Instead of acting as a direct primary elicitor of these cascades, current mechanistic models propose that nanosilicon plays an indirect regulatory role. The experimentally observed upregulation of stress-related transcription factors is hypothesized to result primarily from nanosilicon-mediated ROS modulation. By mitigating primary oxidative damage, nanosilicon alters the cellular redox state. This modulated redox environment can subsequently act as a secondary signal, inferred to influence the activation thresholds of both MAPK cascades and ABA signaling pathways. Therefore, while the downstream transcriptional regulation and the resulting redox homeostasis are confirmed targets of nanosilicon application, its specific entry points into the MAPK and hormone signaling networks should be critically regarded as proposed links that require further targeted molecular investigations.

## 8 Variability across plant species and experimental conditions

The efficacy of nanosilicon in alleviating oxidative stress under salinity conditions exhibits notable variability across plant species and experimental setups. This variability reflects the complex interplay of biological, physiological, and environmental factors influencing nanosilicon’s performance. Distinct species display differently due to inherent variations in their salt tolerance mechanisms, antioxidant capacities, and cellular architectures. For instance, halophytes, which naturally thrive in saline environments, typically display moderate enhancements in ROS scavenging (e.g., 10–20% reduction in H_2_O_2_), following nanosilicon application. Their inherently robust antioxidant systems, characterized by high SOD and CAT activities, may limit additional benefits (Soleimannejad et al., 2019; Abou Baker, 2023). In contrast, glycophytes such as rice (Oryza sativa) and wheat (Triticum aestivum), which are more sensitive to salinity, exhibit pronounced improvements. Controlled studies report reductions in oxidative markers like MDA by up to 50% and boosting antioxidant enzyme activities by as much as 60% (Alharbi et al., 2022; Riaz et al., 2022). This variability is partly due to differences in nanosilicon uptake and translocation, which depend on root morphology, cell wall porosity, and transporter expression. These factors often enable more efficient nanoparticle absorption in glycophytes under stress (Mathur and Roy, 2020; Fan et al., 2022). Experimental conditions further modulate these effects: nanosilicon concentration (e.g., 50–500 mg/L), particle size (e.g., 10 nm *vs*. 50 nm), and application method (foliar spray *vs*. root drenching) significantly alter its impact (Gu et al., 2013; Mathur and Roy, 2020). For example, smaller nanoparticles penetrate tissues more effectively, yielding greater ROS reduction in tomato under high salinity (200 mM NaCl), while higher doses may induce cytotoxicity, negating benefits in sensitive species like Arabidopsis (Fan et al., 2022). Environmental factors such as light intensity, temperature, and salt stress duration also play an important role (Mathur and Roy, 2020), studies under high light report enhanced nanosilicon-mediated protection of photosynthesis in wheat (Khoroshilov et al., 2021), whereas prolonged salinity (e.g., >15 days) can overwhelm its capacity in barley, leading to increased MDA accumulation (or failing to mitigate lipid peroxidation) (Akhter et al., 2022). Soil type and pH further affect nanosilicon solubility and availability, with alkaline soils enhancing its uptake (El-Ramady et al., 2022). These inconsistencies highlight the importance of species-specific and condition-tailored applications. Comparative trials reveal nanosilicon outperforming conventional silicon in rice (Kheyri, 2022) but not in salt-tolerant quinoa under identical setups (Nurbekova et al., 2024). Thus, optimizing experimental parameters is crucial to maximizing nanosilicon’s agronomic potential across diverse crop systems. To provide a clearer overview of how nanosilicon’s efficacy varies depending on the plant type and stress duration, a comparative summary is presented in **Table 4**. This table highlights the differential responses of glycophytes and halophytes, demonstrating that the mitigating effects of nanosilicon are highly dependent on the inherent salt tolerance mechanisms of the species and the severity of the stress.

**Table 4:** Comparative efficacy of nanosilicon (nSi) application across different plant species and salinity conditions.

Plant type/species	Salinity level	nSi application method	Key observations/efficacy
Glycophytes			
Rice (Oryza sativa)	Moderate to High	Foliar/Soil	Up to 50% reduction in MDA; ~60% boost in antioxidant enzyme activity. nSi significantly outperforms bulk Si.
Barley (Hordeum vulgare)	Prolonged (>15 days)	Soil/Hydroponic	Short-term ROS mitigation is effective, but prolonged severe stress overwhelms nSi capacity, leading to eventual MDA accumulation.
*Halophytes*			
Quinoa (Chenopodium quinoa)	High	Foliar/Soil	Modest ROS reduction (10-20%). nSi does not significantly outperform conventional silicon due to the plant’s inherent salt tolerance mechanisms.

## 9 Applications and challenges

### 9.1 Potential of nanosilicon in oxidative stress management

Nanosilicon holds transformative potential for agriculture, particularly in managing oxidative stress under salt stress. Its unique physicochemical properties enable the offer of innovative solutions for enhancing plant tolerance while aligning with sustainable agriculture objectives (Rastogi et al., 2019; Bhat et al., 2021; Etesami et al., 2022). By directly scavenging ROS like O_2_^-^ and H_2_O_2_, combined with its stimulating both enzymatic antioxidants (e.g., SOD, CAT, APX) and non-enzymatic (e.g., glutathione, ascorbate), positions it as a powerful tool to reduce oxidative damage, as evidenced by up to 50% reductions in lipid peroxidation (MDA levels) in crops like wheat and rice under salt stress (Hajihashemi and Kazemi, 2022; Xiong et al., 2024; Dhiman et al., 2021; Ismail et al., 2022; Aghaei et al., 2024). Nanosilicon sustains photosynthetic performance, with up to 35% higher chlorophyll retention reported in nanosilicon-treated maize under salt stress (Alsamadany et al., 2024). Its nanoscale dimensions facilitate delivery to organelles like chloroplasts and mitochondria, improving efficiency compared with bulk silicon (Ali, 2024; Swain et al., 2025). Beyond short-term stress relief, nanosilicon modulates the expression of stress-responsive genes (e.g., SOD, DREB) and interacts synergistically with osmotic and ionic tolerance mechanisms. Field trials show improved yields in tomato and rice by up to 40% (Badawy et al., 2021; Jabal and Abdulkareem, 2022). Low dosage requirements (50–200 mg/L) and potential biodegradability further enhance nanosilicon’s appeal as an eco-friendly alternative to chemical fertilizers or toxic nanoparticles (Gupta et al., 2025; Wahab et al., 2024). In salinized regions, such as arid zones in South Asia and the Middle East, nanosilicon offers a cost-effective strategy to sustain crop productivity and ensure food security (Wang et al., 2022; El-Ramady et al., 2024). Its versatility under combined stresses (e.g., salt and drought) further broadens its applicability across diverse agroecosystems (de Moraes and Lacava, 2022; Okeke et al., 2023).

#### 9.1.1 Field applications of nanosilicon: trials, results, and economic considerations

While most studies on nanosilicon’s role in oxidative stress mitigation have been conducted under controlled greenhouse or pot conditions, emerging field trials demonstrate its practical efficacy in real-world saline agricultural systems, particularly through foliar spray, soil amendment, or seed priming at low doses. These applications leverage nanosilicon’s high bioavailability to reduce ROS levels, enhance antioxidant defenses, and improve yield under natural variability in soil salinity (EC 4–12 dS/m), irrigation quality, and climate (Farhangi-Abriz and Torabian, 2018).

In saline soils, foliar application of nanosilicon to rice and soybean increased grain yield, reduced H_2_O_2_ and MDA by 30–45%, and boosted SOD/CAT activities by 15–60% compared to untreated controls, outperforming bulk silicon by 15–20% (Farhangi-Abriz and Torabian, 2018; Xiong et al., 2024). Field trials showed that nanosilicon enhanced grain yield, preserved chlorophyll content, and lowered lipid peroxidation. Plants grown under salt-affected fields (EC 7–9 dS/m) benefited from seed priming with nanosilicon (50–100 mg/L), resulting in higher grain yield and reduction in Na^+^ uptake, directly linking to reduced oxidative damage. These outcomes highlight nanosilicon’s superiority over conventional silicon in field settings, with yield gains of 20–40% attributed to targeted ROS scavenging and sustained antioxidant enzyme activity under prolonged stress (Awad-Allah, 2023; Omar et al., 2023).

Previous studies reported that nanosilicon was applied through a two-step procedure in which wheat seeds were first soaked in a nanosilicon concentrate before sowing, followed by two foliar applications during the vegetation period, specifically at the tillering and stem elongation stages. Field experiments indicated that nanosilicon treatment improved early seedling performance, increasing germination energy by 18.5% and the final germination rate by 5.5% compared with untreated controls (Khoroshilov et al., 2021). Overall, the nanosilicon application was identified as an effective and low-cost strategy for enhancing the productivity potential of spring wheat.

Previous research also demonstrated that nanosilicon is a more promising option for increasing silicon accumulation in rice tissues than conventional silicon fertilizers, primarily due to its higher bioavailability. These studies further reported that relatively small quantities of nanosilicon fertilizers produced effects comparable to, or even exceeding, those obtained with much larger amounts of traditional silicon sources. This suggests that nanosilicon applications can increase grain yield while simultaneously reducing fertilizer costs. Field trials additionally highlighted that nanosilicon particles outperformed conventional silicon fertilizers by delivering higher crop yield and quality, improving nutrient uptake, and exhibiting superior environmental adaptability (Semina et al., 2020; Qados and Moftah, 2014).

Previous studies described the application method and dosage of nanosilicon in rice field trials, which involved foliar spraying at a concentration of 50 mg L^-^¹ (equivalent to 300 g ha^-^¹) across four distinct growth stages. The field trial results demonstrated that nano-Si treatment significantly increased grain yield by 9.5% compared with the control group, confirming its effectiveness (Kheyri, 2022). Additionally, these studies suggested that the use of nanosilicon, either alone or in combination with nano-zinc, not only enhanced grain yield but also reduced fertilizer costs and minimized environmental pollution relative to conventional soil-applied fertilizers (Kheyri et al., 2019). For comparison, studies utilizing conventional silicon priming, such as sodium silicate seed priming, have demonstrated improvements in plant growth and stress tolerance under saline conditions (Falouti et al., 2025). However, the limited number of direct field comparisons between bulk silicon and nanosilicon treatments highlights the need for further research to accurately quantify their relative efficacy. Overall, these findings indicate that silicon seed priming represents an effective, simple, and low-cost strategy for improving plant productivity in salt-affected agricultural lands.

### 9.2 Limitations and risks of nanosilicon use

Despite many advantages, nanosilicon use in agriculture entails several limitations and potential risks that must be carefully managed (Bhat et al., 2021). Its efficacy is highly dependent on plant species, particle size (e.g., 10 nm *vs*. 50 nm), concentration (e.g., 50–500 mg/L), and application method (foliar *vs*. soil application), often resulting in inconsistent outcomes (Awad-Allah, 2023). This inconsistency is compounded by environmental variables, such as soil pH, salinity levels, and temperature, which further influence nanosilicon’s solubility and bioavailability (El-Ramady et al., 2024). At excessive concentrations, nanosilicon may induce phytotoxicity, as observed in plants where doses above 300 mg L^-1^ increased ROS production rather than mitigating it, highlighting a narrow therapeutic window compared to conventional silicon (Khoroshilov et al., 2021). Additionally, its nanoscale nature raises environmental concerns, including potential accumulation in soil and water systems, and unintended impacts on microbial communities or non-target organisms (El-Ramady et al., 2022). Studies suggest that prolonged exposure has been associated with shifts in soil bacterial diversity, though long-term ecological impacts remain understudied (Rajput et al., 2021; Shi et al., 2025). Concerns also extend to food safety, as nanoparticle residues in food and feed chains could pose human health risks. Regulatory frameworks for nanomaterials in agriculture remain underdeveloped (Kapeleka and Mwema, 2024). On the production side, synthesizing uniform nanosilicon with controlled properties (e.g., size, porosity, and surface charge) is costly and energy-intensive, unlike bulk silicon. Moreover, its interaction with other agrochemicals such as fertilizers and pesticides is poorly understood, potentially leading to antagonistic or synergistic effects (de Moraes and Lacava, 2022). Addressing these challenges will require tailored application strategies, comprehensive toxicity studies, and robust regulatory frameworks to ensure nanosilicon’s safe and effective use (Etesami et al., 2022; de Moraes and Lacava, 2022).

### 9.3 Future directions for research on oxidative stress mitigation

Future research should focus on elucidating nanosilicon’s molecular mechanisms and optimizing its application to unlock its full agricultural potential. First, gaining mechanistic insights is a crucial priority. This involves investigating how nanosilicon influences redox-sensitive signaling pathways (e.g., MAPK, ABA) and epigenetic modifications (e.g., DNA methylation, histone acetylation). Integrative omics approaches, such as transcriptomics and proteomics, could reveal species-specific regulatory networks in glycophytes versus halophytes like *Salicornia*. Furthermore, formulation optimization requires assessing the effects of particle size (e.g., 5 nm *vs*. 50 nm), surface functionalization (e.g., with amino or hydroxyl groups), and delivery methods (e.g., nanoemulsions *vs*. soil amendments) on ROS scavenging efficiency and phytotoxicity.

Another critical area is understanding the environmental fate of these nanoparticles by evaluating nanosilicon’s long-term degradation, accumulation, and impact on soil microbiota through field-scale trials. Additionally, exploring integrated strategies is highly recommended. Combining nanosilicon with other stress mitigation strategies, such as biofertilizers, conventional silicon, or genetic engineering (e.g., overexpressing SOD), can enhance synergistic effects—for example, achieving a 60% MDA reduction in tomatoes under high salinity. However, it is imperative to acknowledge that any integration with genetic engineering must carefully navigate stringent regulatory frameworks and address potential challenges regarding public acceptance of genetically modified crops.

Concurrently, ensuring safety and complying with regulations are paramount; this necessitates conducting toxicological assessments of nanosilicon residues in edible tissues and establishing nanomaterials-specific agricultural guidelines. Finally, utilizing predictive modeling through artificial intelligence and simulation tools will be valuable to predict nanosilicon performance under complex stresses (e.g., salt + drought). Together, these directions will refine nanosilicon’s role in oxidative stress mitigation, ensuring it contributes effectively to sustainable agriculture in an era of increasing salinization.

**Figure 2:**
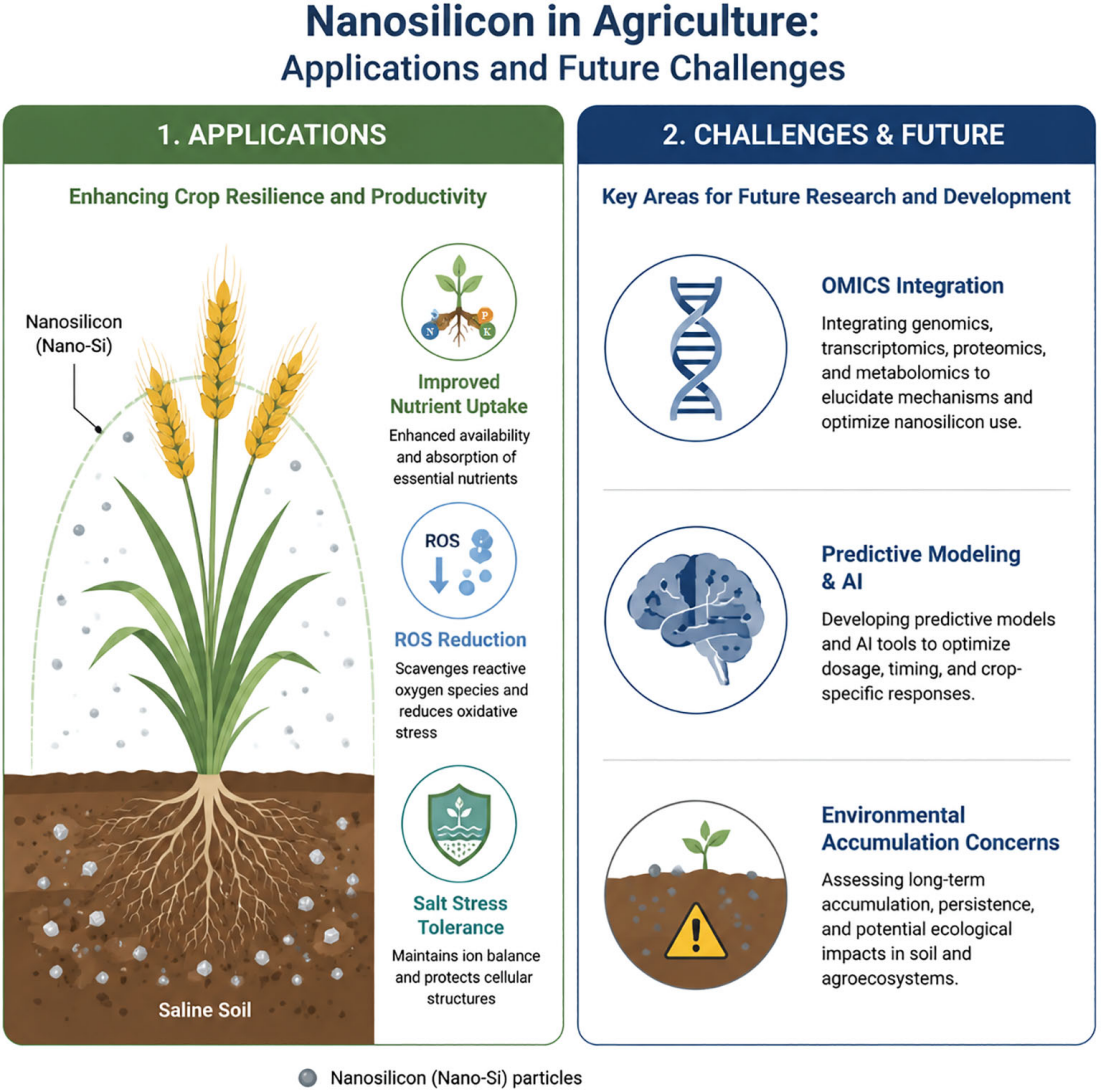
Overview of the current applications and future perspectives of nanosilicon in agriculture. The left panel highlights the primary agronomic benefits of nanosilicon, particularly in enhancing crop resilience under saline conditions through improved macronutrient uptake, mitigation of oxidative stress via ROS reduction, and overall augmentation of salt stress tolerance. The right panel outlines critical challenges and future research frontiers necessary for sustainable application. These include the integration of multi-omics approaches to unravel complex molecular mechanisms, the utilization of artificial intelligence (AI) and predictive modeling for optimizing application protocols, and the crucial need for comprehensive ecological risk assessments regarding the long-term accumulation and environmental impact of nanomaterials in agroecosystems.

Beyond elucidating the specific cellular protective mechanisms, it is essential to contextualize the broader role of nanosilicon in modern agricultural systems and outline the trajectory for future research (**Figure 2**). The practical applications of nanosilicon are primarily driven by its capacity to significantly enhance crop resilience and productivity, especially under adverse environmental conditions such as soil salinity. As depicted, the application of nano-Si bolsters plant vigor by facilitating the efficient absorption of essential nutrients (nitrogen, phosphorus, and potassium), scavenging stress-induced ROS, and preserving structural and ionic integrity during salt stress. However, to transition these benefits into widespread and sustainable agricultural practices, several critical challenges must be addressed. Future research endeavors must prioritize the integration of advanced multi-omics technologies, encompassing genomics, transcriptomics, proteomics, and metabolomics, to thoroughly decipher the intricate molecular networks modulated by nanosilicon. Furthermore, the development of artificial intelligence (AI) and predictive modeling tools will be indispensable for optimizing precision agriculture, allowing for the precise calibration of dosages and application timings tailored to specific crop species and environmental variables. Ultimately, as the deployment of nanomaterials expands, rigorous and continuous ecological assessments are imperative to monitor the long-term accumulation, persistence, and potential toxicological impacts of nanosilicon within soil matrices and broader agroecosystems.

## 10 Conclusion

Nanosilicon emerges as a potent and versatile tool for mitigating oxidative stress in salinity-stressed plants. Its small size, high specific surface area, and reactivity enable it to directly scavenge ROS, especially through reactive silanol groups on the nanoparticle surface. In parallel, nanosilicon enhances both enzymatic and non-enzymatic antioxidant systems, reducing oxidative damage and protecting vital cellular structures. Studies have shown that nanosilicon application can lead to significant reductions in MDA levels, indicating lower lipid peroxidation, and increases in antioxidant enzyme activities such as SOD, CAT, and POD. Moreover, nanosilicon can modulate stress-responsive genes via ABA and MAPK signaling pathways, contributing to enhanced osmotic and ionic tolerance. Its effect on improving chlorophyll content, membrane integrity, and ion homeostasis further underscores its potential as a sustainable alternative to traditional inputs in salinity management. However, it is crucial to emphasize that the successful application of nanosilicon is not universal; its efficacy is highly dependent on plant species, nanoparticle size, applied concentration, and specific environmental conditions. Potential phytotoxicity and environmental risks, as well as economic feasibility, need careful consideration before widespread adoption.

To bridge the gap between current knowledge and field application, future research must prioritize several key areas. The top research priorities include elucidating the precise molecular mechanisms of nSi-plant interactions (particularly regarding redox-sensitive signaling and epigenetic modifications), optimizing nanoparticle formulations for specific crop needs, and conducting comprehensive, long-term field evaluations to assess its environmental fate and ecological safety. Ultimately, integrating these empirical findings with predictive AI modeling and advanced breeding strategies will be essential for developing scalable, scientifically robust, and sustainable applications of nanosilicon in salinity-affected agricultural systems.
